# An integrated modelling methodology for estimating global incidence and prevalence of hereditary spastic paraplegia subtypes SPG4, SPG7, SPG11, and SPG15

**DOI:** 10.1186/s12883-022-02595-4

**Published:** 2022-03-24

**Authors:** Geert Vander Stichele, Alexandra Durr, Grace Yoon, Rebecca Schüle, Craig Blackstone, Giovanni Esposito, Connor Buffel, Inês Oliveira, Christian Freitag, Stephane van Rooijen, Stéphanie Hoffmann, Leen Thielemans, Belinda S. Cowling

**Affiliations:** 1 Integrated Strategic Market Access Services (ISMS), Rodendijk 60Y, 2980 Zoersel, Belgium; 2 GenBytes, Schoondreef 7, 2330 Merksplas, Belgium; 3 Sorbonne Université, Paris Brain Institute, Paris, France; 4 Divisions of Neurology and Clinical and Metabolic Genetics, The Hospital for Sick Children, University of Toronto, Toronto, Canada; 5 Hertie Institute for Clinical Brain Research, Tubingen, Germany; 6 Department of Neurology, Massachusetts General Hospital, Boston, MA USA; 7 Dynacure, 67400 Illkirch, France; 8 2 Bridge, Rodendijk 60/X, 2980 Zoersel, Belgium

**Keywords:** Hereditary spastic paraplegia, Epidemiology, Epidemiological model, Prevalence, Incidence

## Abstract

**Background:**

Hereditary spastic paraplegias (HSPs) are progressively debilitating neurodegenerative disorders that follow heterogenous patterns of Mendelian inheritance. Available epidemiological evidence provides limited incidence and prevalence data, especially at the genetic subtype level, preventing a realistic estimation of the true social burden of the disease. The objectives of this study were to (1) review the literature on epidemiology of HSPs; and (2) develop an epidemiological model of the prevalence of HSP, focusing on four common HSP genetic subtypes at the country and region-level.

**Methods:**

A model was constructed estimating the incidence at birth, survival, and prevalence of four genetic subtypes of HSP based on the most appropriate published literature. The key model parameters were assessed by HSP clinical experts, who provided feedback on the validity of assumptions. A model was then finalized and validated through comparison of outputs against available evidence. The global, regional, and national prevalence and patient pool were calculated per geographic region and per genetic subtype.

**Results:**

The HSP global prevalence was estimated to be 3.6 per 100,000 for all HSP forms, whilst the estimated global prevalence per genetic subtype was 0.90 (SPG4), 0.22 (SPG7), 0.34 (SPG11), and 0.13 (SPG15), respectively. This equates to an estimated 3365 (SPG4) and 872 (SPG11) symptomatic patients, respectively, in the USA.

**Conclusions:**

This is the first epidemiological model of HSP prevalence at the genetic subtype-level reported at multiple geographic levels. This study offers additional data to better capture the burden of illness due to mutations in common genes causing HSP, that can inform public health policy and healthcare service planning, especially in regions with higher estimated prevalence of HSP.

**Supplementary Information:**

The online version contains supplementary material available at 10.1186/s12883-022-02595-4.

## Background

Hereditary spastic paraplegias (HSP) comprise a group of inherited neurodegenerative disorders with heterogeneous clinical and genetic manifestations. HSP-related symptoms are associated with impairment of the longest corticospinal axons, predominantly presenting as bilateral progressive spasticity of the lower limbs in affected patients [1–3]. HSP is often characterized clinically as pure (uncomplicated), or complex (complicated) forms. In pure forms, patients present clinical characteristics that include progressive lower extremity spastic weakness, mild diminution of distal vibratory sensation, and urinary bladder symptoms [1, 2, 4]. In complex forms, additional clinical features are also observed, such as cerebellar ataxia, seizures, intellectual disabilities, visual changes, parkinsonism, cognitive impairment, and peripheral neuropathy [1, 4, 5].

HSPs are most frequently inherited in an autosomal dominant pattern [1, 2, 4]. The most common subtype of AD-HSP, SPG4, is caused by pathogenic variants in the *SPAST* gene, encoding spastin. The most frequently reported symptoms of SPG4 include lower limb spasticity, hyperreflexia of the lower and sometimes upper limbs, in addition to extensor plantar responses, decreased vibratory sensation, bladder disturbance, and muscle weakness [1, 4, 5]. In contrast, patients with the most common form of autosomal recessive HSP, due to pathogenic loss-of-function mutations in *SPG11*, encoding spatacsin*,* have a more severe course with complications that can include thinning of the corpus callosum, CNS white matter changes, early-onset parkinsonism and ataxia, and intellectual disability [1, 2, 4, 6]. Other AR-HSP patients also show biallelic mutations in *SPG5*, *SPG7*, *SPG15*, *SPG35*, and *SPG54*, with overall mutation frequencies of 13 and 7% for SPG7 and SPG15, respectively [1].

Age at onset within and across genetic subtypes of HSP is variable, ranging from the first to the eighth decade [1, 5, 6]. These data reflect both biological variability and reporting inaccuracy of slowly progressing disease. Although the distribution is bi-modal, the mean age at onset for HSPs is around 31 years [6].

The current epidemiology reports provide limited incidence and prevalence data since the association between genetic subtypes and clinical phenotypes remains incomplete due to small sample size or restricted studies, which, in turn, do not allow for a realistic estimation of the social burden of the disease, especially at the genetic subtype level. Recently, a clear phenotype-genotype correlation has been published, with de novo missense pathogenic variants in *SPAST* responsible for early onset of SPG4 [5].

To address these challenges, we developed a model using available evidence to obtain a better estimate of global HSP patient numbers by age, causative gene, symptom onset, and geographic regions, including at the country level. We selected the most common mutations with data available, for both AD-HSP and AR-HSP (genetic subtypes SPG4, SPG7, SPG11, and SPG15), and divided the population into pre-symptomatic (before reaching age at onset) and symptomatic (after reaching age at onset). In this model, we focused on specific countries of interest, where the patient pool was estimated for each of these populations. Countries of interest included those where HSP prevalence data was available (Denmark, Japan), North America (Canada, US), EU4 countries (France, Germany, UK, Italy), and Belgium. In addition, country and region-level estimates were explored for all other countries but with less detail.

This more comprehensive model will allow the drug developers to make evidence-based decisions with additional insights on the natural course of the disease over time, and the impact at the country or regional-level. Additionally, it will raise awareness of the true burden of illness across the spectrum of this disease and better target unmet needs, maximizing societal benefit. Finally, this will support health care service planning for those impacted by this disease.

## Methods

### Logic of the model

**Fig. 1:**
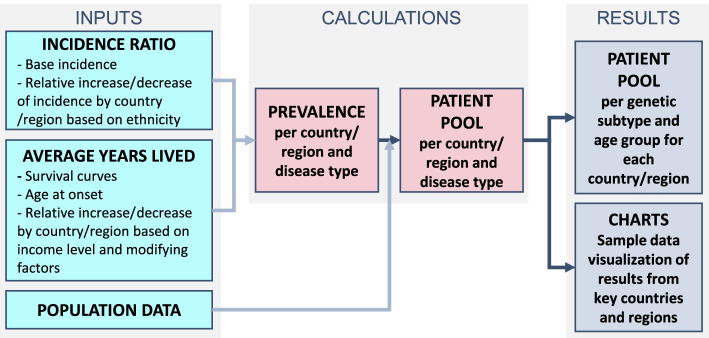
Model Logic Flow. Schematic overview of the methodology to estimate key measures. Step 1: collect information on incidence ratio and average years lived. Step 2: determine prevalence per country/region and disease type. Step 3: using additional population data, determine the patient pool per country/region and disease type. Step 4: estimate the patient pool per genetic subtype and age group for each country/region. Step 5: sample data visualizations of results from key countries and regions

In order to capture HSP prevalence, estimates of both incidence and survival data are required for the different genetic subtypes. Incidence can be determined by combining available prevalence data, mutation frequency, and survival curves (based on severity) [7]. Because of the limited data availability, heterogeneity and variability related to rare diseases, the source data were collected and structured hierarchically according to data reliability and grouped in published direct evidence, published indirect evidence, and data with limited published evidence or limited patient numbers. Estimates of incidence at birth and survival data were used to capture HSP patient prevalence by HSP genetic subtype, age, and geographies (Fig. 1).

### Epidemiological assessment

To define key epidemiological parameters – prevalence, incidence at birth, survival, and mutation frequency of HSP genetic subtypes across sub-population, a targeted literature search was performed, and expert opinion was provided from four physicians with expertise in HSP (HSP Experts), located in US, France, Germany, and Canada.

The base selection included two meta-analyses, one from Omidvar et al. that compiled 147 published studies with 13,570 HSP patients [1], and another from Ruano et al. that reviewed 22 studies, reporting on 14,539 patients from 16 countries [2]. Additionally, a search was conducted on PubMed for HSP studies published after 2013 using the search terms Strumpell-Lorrain OR (spastic paraplegia) OR (hereditary spastic paraplegia) OR (SPG11) OR (SPG4) OR (SPAST)) AND ((epidemiology) OR (prevalence) OR (incidence) OR (mortality) OR (survival)) AND ((humans[Filter]) AND (2013:2020[pdat])). The 89 studies published after 2013 were assessed for the predetermined inclusion criteria (study design: population-based prevalence studies, course of disease, newborn screening, or reporting data on: demographics, patient distribution, mutation frequency). Seventy-six studies were excluded for the following reasons: did not match inclusion criteria, duplicates (already captured in Ruano et al. review), or reported on HSP genetic subtypes that were not of primary interest. Of 13 studies selected, two studies were included for population-based prevalence, two studies for the course of disease, seven studies for demographic and patient distribution, and two reviews covering several inclusion criteria. No studies were found related to newborn screening. Furthermore, six studies were identified by HSP Experts and integrated into the body of evidence [3, 5, 8–11]. A quality assessment was performed on available data using the following characteristics: number of cases, population assessed and patient identification strategy, and study time period. The studies considered in this model are included in Supplementary Table 2.

### Model parameters

Using the best available evidence, model parameters were extracted and/or generated using indirect evidence. These parameters were used to populate the model and output was validated by HSP Experts. In general, available data on prevalence, mutation frequency, survival, and age at disease onset (symptom onset) were used to generate duration of illness (years lived with symptoms after onset), incidence at birth, and, in turn, prevalence for each genetic subtype. To generate model outputs at the country and regional levels, incidence at birth was also corrected using modifying factors accounting for ethnic differences. A detailed description of the estimations is provided per model parameter in subsequent sections.

### Estimation of survival

The survival probability at time t was calculated as.

$$\mathrm{S}\left(\mathrm{t}\right)=\mathrm{S}\left(\mathrm{t}-1\right)\ast \left(1-{\mathrm{N}}_{\mathrm{deaths}}/{\mathrm{n}}_{\mathrm{at}\ \mathrm{risk}\ \mathrm{at}\ \mathrm{beginning}\ \mathrm{of}\ \left(\mathrm{t}\right)}\right)$$

where the number of deaths was estimated as.

n of patients at t-1 – n of patients at t.

The survival curves were then used to estimate the life expectancy for HSP patients by measuring the area under the curve using the Trapezoid rule [12].

$$\mathrm{Life}\ \mathrm{expectancy}={\Sigma}_{\left(\mathrm{i}\ \mathrm{to}\ \mathrm{k}\right)}\ \left({\mathrm{t}}_{\mathrm{i}+1}-{\mathrm{t}}_{\mathrm{i}}\right)\ast \left(\mathrm{S}\left({\mathrm{t}}_{\mathrm{i}+1}\right)+\mathrm{S}\left({\mathrm{T}}_{\mathrm{i}}\right)\right)/2$$

k = number of age intervals; i = index

For SPG11 and SPG15, the survival curves were adjusted based on HSP expert input that patient survival was impacted starting at around the mid-thirties. Therefore, a linear Hazard Ratio (HR) was applied to the general population survival curve, increasing between the ages of 35 and 60, followed by a constant HR from the age of 60 years onward (Supplementary Table 1). The HR increase was calibrated to have a minimal impact on the life expectancy years that were calculated from the HSP survival curve. Therefore, the survival probability for SPG11 and SPG15 was calculated combining the survival probability of the general population and applying a HR using:

$$\mathrm{S}\left(\mathrm{SPG}11,-15\right)=\mathrm{S}\left(\mathrm{General}\ \mathrm{population}\right)\hat{\mkern6mu} \mathrm{HR}$$

The HR was additionally calibrated so that the life expectancy would align with calculations based on available data suggesting a life expectancy of around 74 years.

The survival curves obtained for high-income countries were then adjusted for country income level, using the additional UN definitions (Upper Middle Level, Lower Middle Level, and Low Level). In turn, the survival ratio between HSP patients and the general population was assumed constant. The survival curves were thus used to estimate life expectancy for HSP patients across countries with different income level by measuring the area under the curve using the Trapezoid rule [12].

### Estimation of incidence

The incidence at birth was calculated from prevalence and life expectancy (including symptomatic years) as follows:

$$\mathrm{Incidence}\ \mathrm{at}\ \mathrm{birth}\ \mathrm{per}\ 100\;\mathrm{k}=\mathrm{prevalence}\ \mathrm{per}\ 100\;\mathrm{k}/\left(\mathrm{Ys}/\mathrm{Ygp}\right)$$

Ys = years symptomatic; Ygp = life expectancy in years of the general population.

Since prevalence data for SPG7 and SPG15 were not available, the prevalence per 100,000 was calculated as a proportion of SPG11 prevalence using the mutation frequency reported previously [1]. The prevalence per 100,000 was thus calculated as 0.19 and 0.10 per 100,000, for SPG7 and 15 respectively.

Relative incidence ratio across regions was calculated by grouping countries in 9 regions according to the UN classification. For each region, the ethnic composition was estimated as the average composition of the most populated countries in that region, and all the countries in a particular region were assumed to have the average region composition (Supplementary Table 3). For the specific countries of interest, the ethnic composition was estimated more accurately using data available for those countries (Supplementary Table 4).

## Results

### Review of available data

#### AD- and AR-HSP subtype prevalence from population studies

**Fig. 2:**
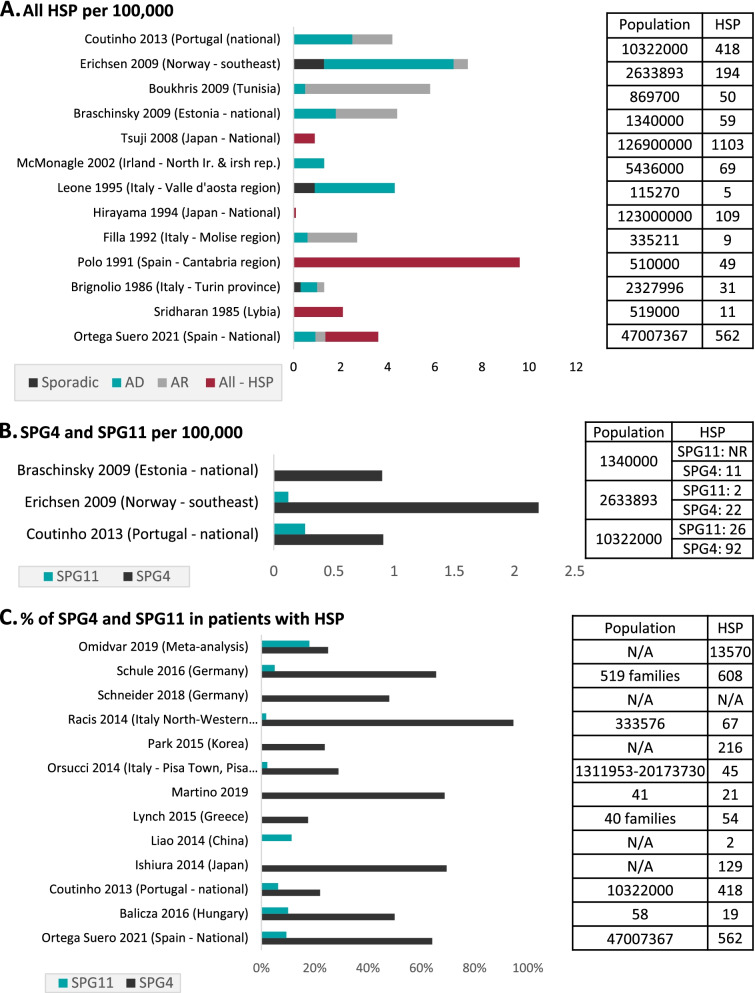
Reported population-based prevalence. **a** Prevalence of Sporadic, AD, AR, and all HSP cases per 100,000 in Ortega Suero et al. 2021, and in key studies assessed in Ruano et al. 2014. Total population assessed, and number of HSP cases are represented on the right. **b** Reported SPG4 and SPG11 prevalence per 100,000 in Estonia, Norway and Portugal based on detailed genetic analysis. Total population assessed and number of HSP cases, divided by SPG4 and SPG11, are reported on the right. **c** Reported proportion of SPG4 and SPG11 in all HSP in selected studies

Data on prevalence, patient distribution, and age at onset were available for diagnosed HSP patients. However, direct incidence and survival rates data were not. Sources of available prevalence data, which are described per study in Supplementary Table 5, used various methodologies and data sources to assess prevalence in different countries, most of which reported on diagnosed HSP individuals only. Accordingly, the available parameters were used to estimate missing epidemiological parameters (Fig. 2a).

Ruano et al. reported studies, mainly from Asian and European populations, and found that prevalence was highly variable, reflecting population differences and methodological heterogeneity, such as inclusion, diagnosis, and classification of patients [2]. The pooled average combined prevalence for AD-HSP and AR-HSP was 1.8/100,000 and, when considering only studies after 2000 (when genetic testing was more broadly available), the prevalence for both AD-HSP and AR-HSP increased to 2.2/100,000 [2], in alignment with the published prevalence of 2.24 cases per 100,000 inhabitants identified in Spain [13] (Fig. 2a).

For AD-HSP, prevalence ranged from 0.5–5.5/100,000 [14–21], with the highest value reported in southeast Norway, potentially due to isolation or a founder effect. For AR-HSP, the prevalence ranged from 0.3–5.3/100.000 [14–16, 18–21], with the highest prevalence found in Tunisia, possibly due to a higher level of consanguinity in the Arabic population. An additional seven studies, not previously included in the meta-analyses, also reported data on prevalence consistent with the findings of Ruano et al. [22–27, 43].

These studies already highlight differences at the country level, which are influenced by consanguinity, isolation effects, access to genetic testing, methodological differences in case finding, and study inclusion/exclusion criteria [2]. Additionally, due to the inherent challenges of epidemiological studies in rare diseases, there are some key limitations in the systematic literature reviews and meta-analyses of population-based data in HSP. This is especially the case when evaluating at country and regional levels, as large areas of the world remain without prevalence studies, like the Americas, Africa, Southeast Asia, and Oceania.

#### Prevalence of genetic subtypes across countries

The extent of data available differed among genetic subtypes, with more data available for SPG4 and SPG11 (with a total of 18 studies across 12 countries [1, 6, 18, 19, 21–23, 28–35]), and less data available for the less common variants – SPG7 and SPG15. Specifically, seven studies in six countries were identified that covered SPG7, and only three studies in three countries reported data on SPG15 (Fig. 2b). For SPG4 and SPG11, available data were representative of populations from Norway and Portugal, where the prevalence of SPG4 per 100,000 (2.2 and 0.91, respectively) was higher than the prevalence of SPG11 per 100,000 (0.12 and 0.26, respectively), and was variable between countries [19, 21]. Additionally, several other studies reported prevalence data on SPG4 in Estonia, Germany, South Korea, Greece, Italy, and Japan, however prevalence data for SPG11 has not been reported [18, 28, 29, 31, 34, 35]. The ratio of AR-HSP to AD-HSP generally ranged from 0.7 to 19.7, as reported in five studies [14, 18, 19, 21, 22] covering Tunisia, Estonia, Portugal, Norway, and Italy.

#### Relative frequency of genetic subtypes

Data were available on the prevalence of SPG4 from 13 studies in 10 countries [1, 6, 18, 19, 21–23, 28, 29, 31, 32, 34, 35], where the frequency of SPG4 relative to HSP ranged from 18% in Greece [29] to 95% in Italy-Sardinia [22]. The frequency of SPG4 relative to AD-HSP ranged from 25% (global) to 61% in Germany [6]. Additionally, a study from Japan reported that SPG4 represented 6% of all sporadic cases [31] (Fig. 2c). Comparing frequencies of SPG4 and SPG11 showed that, overall, SPG4 prevalence was 5–12× greater than SPG11 in Europe and Asia, with the highest frequency of SPG11 in China [36].

**Fig. 3:**
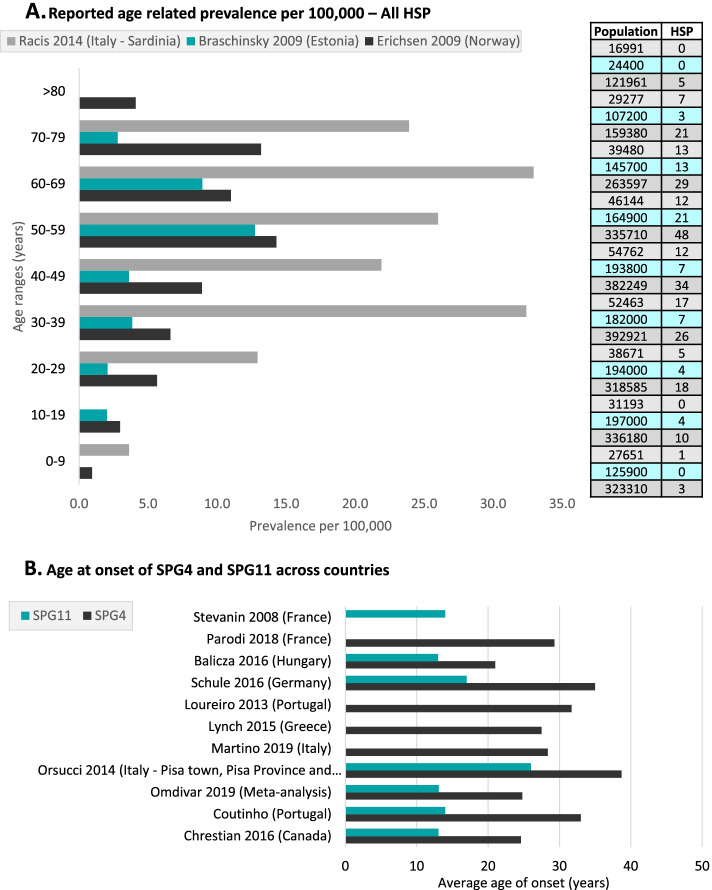
Reported age-related prevalence of HSP. **a** Reported age-related prevalence of all HSP cases per 100.000, based on three studies from Italy, Estonia and Norway. Total population and number of HSP cases identified are reported on the right. **b** Reported average ages at onset in years of SPG4 and in SPG11, based on studies from Hungary, Germany, Portugal Greece, Italy, Canada, and in a worldwide review

A meta-analysis reported that, globally, 18% of HSP patients had *SPG11* gene mutations, based on 27 studies [1]. Furthermore, data were available on the prevalence of SPG11 from seven studies in six countries [1, 6, 22, 23, 29, 32, 33], where the mutation frequency of SPG11 in HSP patients ranged from 2% in Italy-Pisa/Tuscany [23] to 11% in China [30]. The frequency of SPG11 relative to AR-HSP was highly variable, ranging from 8 to 20% at the country-level [19, 21, 33], and up to 50% in a previous review [4] (Fig. 3a).

The frequency of SPG7 relative to total HSP patients was reported in 7 studies, ranging from 4 to 23%, and the global frequency was 13% according to a meta-analysis [1]. For SPG7, the frequency in relation to SPG11 was determined as 72%, and for SPG15 relative to SPG11 was 38%.

SPG15 frequency relative to total HSP patients was reported in 5 studies, ranging from 1 to 15% [1]. The consolidated frequency generated from a meta-analysis was 7%. Additionally, a relative frequency could be calculated from the prevalence data of a Portuguese national study, where it was found to represent 0.7% of total HSP cases [19].

#### Frequencies of genetic subtypes in ethnic subpopulations

As reported by Omidvar et al. [1], the frequency of SPG4 in HSP patients was higher in the Asian population than among Caucasians and Americans (32.62% compared to 23.07 and 24.83%, respectively), although it was considered to be a non-significant difference (*Q* = 3.47, *P* = 1.00). The authors defined ‘American’ as populations from North and South America and ‘Caucasian’ as populations from all or parts of Europe, Western Asia, Central Asia, South Asia, North Africa, and the Horn of Africa. The same trend was observed for SPG11, with a frequency of 87.98% in HSP patients from Asian populations, compared to Caucasians (10.55%), and Americans (24.23%). Lastly, SPG7 was reported with a frequency of 13.29 and 18.97% in Caucasian and American HSP patients, respectively. No data was reported for SPG15 frequency by ethnic group.

#### Age at onset & prevalence per age group and subtype

HSP prevalence per age group was available in four studies from four countries [17, 18, 21, 22]. The most common age group reported with HSP was 50–69 years in an Estonian population [18]. In Italy, a bimodal distribution was observed, with peaks between 30 and 39 and 60–69 years [22]. An increasing prevalence with older age reported in Norway [21] also supported the notion of late clinical manifestations and a (near) normal life expectancy (Fig. 3a). A registry study of 609 HSP patients also reported the age at onset distribution across all HSPs, where a bimodal distribution was also noted with a first peak in childhood and a second peak around 40 years of age [6].

A few studies reported prevalence of HSP genetic subtypes and genotypes per age group. AD-HSP prevalence per age group was available in two studies from two countries (Ireland and Norway). In those studies, AD-HSP was found to be more prevalent in those aged 40+ years than in younger age groups [17, 21]. However, in the Irish study, the prevalence was lower than reported previously and, according to the authors, this difference might be due to methodological differences such as focusing only on “pure” AD-HSP cases [17]. AR-HSP prevalence per age group was only reported in Norway, with the highest prevalence among 20–29-year-olds, and no patients reported under 20 nor above 70 years old. Additionally, an earlier onset was associated with a more severe disease course [21]. Sporadic-HSP prevalence per age group was also only reported in Norway. The study found that Sporadic-HSP was more prevalent in patients over 50 years old and, more specifically, between 60 and 70 years old [21].

Based on the Omidvar et al. meta-analysis, the mean age at onset for each genetic subtype was estimated at 24.8, 13.1, 37.2, and 14.7 years, for SPG4, SPG7, SPG11, and SPG15, respectively [1] (Fig. 3b). The values for SPG4 and SPG7 were comparable with recent studies in France and Europe, which reported mean ages at onset of 29.3 and 35.5, respectively [1, 5, 8]. Although age at onset differed across the genetic subtypes studied, it also varied among patients regardless of the type of mutation [2]. For example, the mean age at onset was highly variable for SPG4, ranging from 21 to 38 years old across 13 studies (Supplementary Table 6). However, it presented a bimodal distribution, occurring between birth and the first decade of life, and between the third and fifth decades [5]. A later age at onset in SPG4 was associated with more severe manifestations, also contributing to more reports and diagnosed forms later in life [37]. A similar phenomenon was identified with SPG11, with reported ages at onset varying from 13 to 26 years old [1, 5, 8].

Genetic subtype data was available across ethnicities for SPG4 and SPG11, but not for the other genetic subtypes assessed in this study. In particular, Omidvar et al. [1] derived the mean age at onset of SPG4 across subpopulations, which varied from 21.88 years in Caucasians, to 28.85 in Asians, and to 30.97 in Americans. For SPG11, sub-group analysis by Omidvar et al. revealed that the mean age at onset was 13.09 years in the Caucasian population, 12.76 years in the Asian population, and 12.79 years in Americans [1]. In line with earlier onset, patients with SPG11 mutations are generally younger than SPG4 patients, and present limited differences among ethnicities. SPG4 shows an earlier onset in Caucasians and Asians, compared to the American population.

### Estimation of incidence at birth & survival

**Fig. 4:**
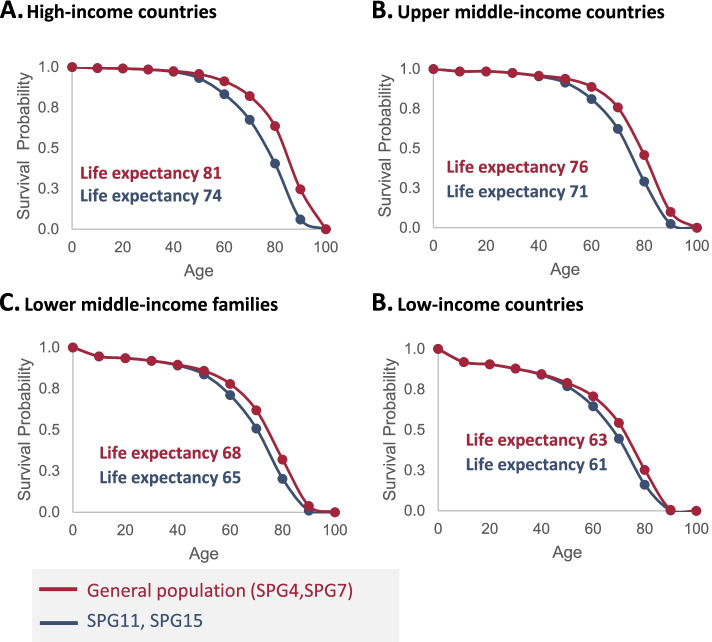
Modelled survival curves across countries. Survival curves for high- (**a**), upper middle-(**b**), lower middle-(**c**), and low-income (**d**) countries, based on UN income level. Red: general population, SPG4 and SPG7; blue: SPG11 and SPG15

To model the HSP pool data on incidence at birth and survival are required. Direct evidence of HSP patients’ survival rate was not available, therefore, survival curves were estimated based on indirect evidence (prevalence data by age group) and HSP expert advice. To estimate an overall HSP survival curve, we used prevalence data by age group present in three studies [18, 21, 22]. In accordance with previous findings, these results showed that the HSP prevalence per 100,000 increased from ages 10 to 60 years, which is compatible with a later onset of the disease. Moreover, this also suggests that the mortality rate of HSP patients until age 60 years is similar to that of the general population. Based on this observation, a survival curve was estimated using the average number of patients from the three studies, and the survival of HSP patients until 60 years of age was assumed to be equal to that of the general population, using data (life expectancy per age group) from the United Nations (UN) for high income countries [38]. According to HSP experts, SPG4 and SPG7 patients do not typically have reduced life expectancy, and therefore the survival curves were consistent with the general population data from UN high income countries (Fig. 4). For SPG11 and SPG15, the survival curves were adjusted to reflect reduced lifespan starting from mid-thirties. Life expectancy ranged from 60 (low-income countries) to 73 (high-income countries).

To estimate the incidence at birth for each genetic subtype, age at onset was used to calculate the duration of illness (years lived with symptoms). The incidence at birth for the base-case was calculated for SPG4, SPG7, SPG11, and SPG15, and was, respectively, 1.24, 0.34, 0.35, and 0.14 per 100,000 (see methods section for formula). We next looked at incidence across different regions. Taking into account the mean age at onset for each genetic subtype studied and the different prevalence among subpopulations, we expected to obtain distinct values for the incidence at birth across different subpopulations. To estimate the relative incidence ratio in different subpopulations, countries were grouped in 9 regions according to the UN classification: Sub-Saharan Africa, Northern Africa, and Western Asia, Central and Southern Asia, Eastern and South-eastern Asia, Latin America and the Caribbean, Australia/New Zealand, Oceania (except Australia and New Zealand), Europe, and Northern America. The average years lived were therefore adjusted at the country-level based on the ethnic composition assessed.

The risk factors, such as consanguinity and founder/isolation, affecting age at onset in the reported subpopulations were calculated using the data from Omidvar et al. [1], and were validated by HSP experts. These factors were used in the model to adjust incidence at birth and average years lived at the country-level, considering the distribution of the modifying factors (Supplementary Tables 1, 4). For SPG4, reported data indicated that the mean age at onset varied across subpopulations. Therefore, ethnicity data from Omidvar et al. [1] was used to adjust age at onset using relative incidence ratios of 0.88, 1.16, and 1.25 for Caucasians, Asians, and Americans, respectively.

For the countries and regions with available data, the relative distribution of AD and AR by geography helped to quantify modifying factors for the incidence at birth, such as isolation/founder effect and consanguinity frequency. Considering Coutinho, 2013 [19] and Braschinsky, 2009 [18] studies as base- case, where the effects of these factors were considered minimal, a split 50–50% for AD and AR was estimated. Deviations from the base-case were identified in Norway [21] and Sardinia [22], where AD was about 13× higher, and in Tunisia [14] where AR was about 10× higher. The analyzed data suggested an increased risk of AD-HSP in Norwegian and Sardinian populations, likely attributed to a founder effect, and an increased risk of AR-HSP in North Africa and Western Asia, likely due to higher consanguinity. This was consistent with conclusions of Ruano et al. [2].

To account for the founder effect, an increase in AD-HSP incidence risk was estimated as the ratio between AD/AR ratio in the Sardinian and Norwegian subpopulations versus Caucasians, considered as the reference population. A factor of 13 was calculated, but a more conservative risk factor of 10 was applied to SPG4. The estimated founder effect of 2% was assigned to each country/region based on the reported data from Norway and Sardinia, where 2% of the population investigated was affected in available studies [21, 22]. For the recessive forms -- SPG11, SPG7, and SPG15 -- the relative ratio of 10 was assigned to the Arabic subpopulation in each key country/region, based on the data from Tunisia (13× greater). This factor was halved for Arabic immigrants in non-Arabic countries, where the consanguinity in the immigrant population was assumed to be reduced. This phenomenon has been demonstrated in a Pakistani population in Norway, which reported a 39% reduction in first cousin marriage among Norwegian Pakistanis compared to those born in Pakistan [39].

We next estimated the age of onset of symptoms. For HSP, the average years with symptoms were calculated from the age at onset reported by Omidvar et al. [1]. This estimation was attributed to the Caucasian subpopulation, since the prevalence data used was reported in studies from Portugal and Estonia. A previously described bimodal distribution was also identified for SPG4 [5]. By integrating the average years symptomatic with the life expectancy reported for SPG4, it was possible to calculate the average number of years that patients live with a pre-symptomatic status. For SPG4, patients were calculated to live, on average, 24.7 years without symptoms, followed by 56.1 years with symptoms. For SPG11, SPG7, and SPG15, the same approach was followed, resulting in average years lived of 13.0, 36.8, and 14.6 without symptoms, and 60.6, 44.0, and 59.0 with symptoms, respectively (Fig. 4).

### Modelling of HSP prevalence per subtype and region

**Fig. 5:**
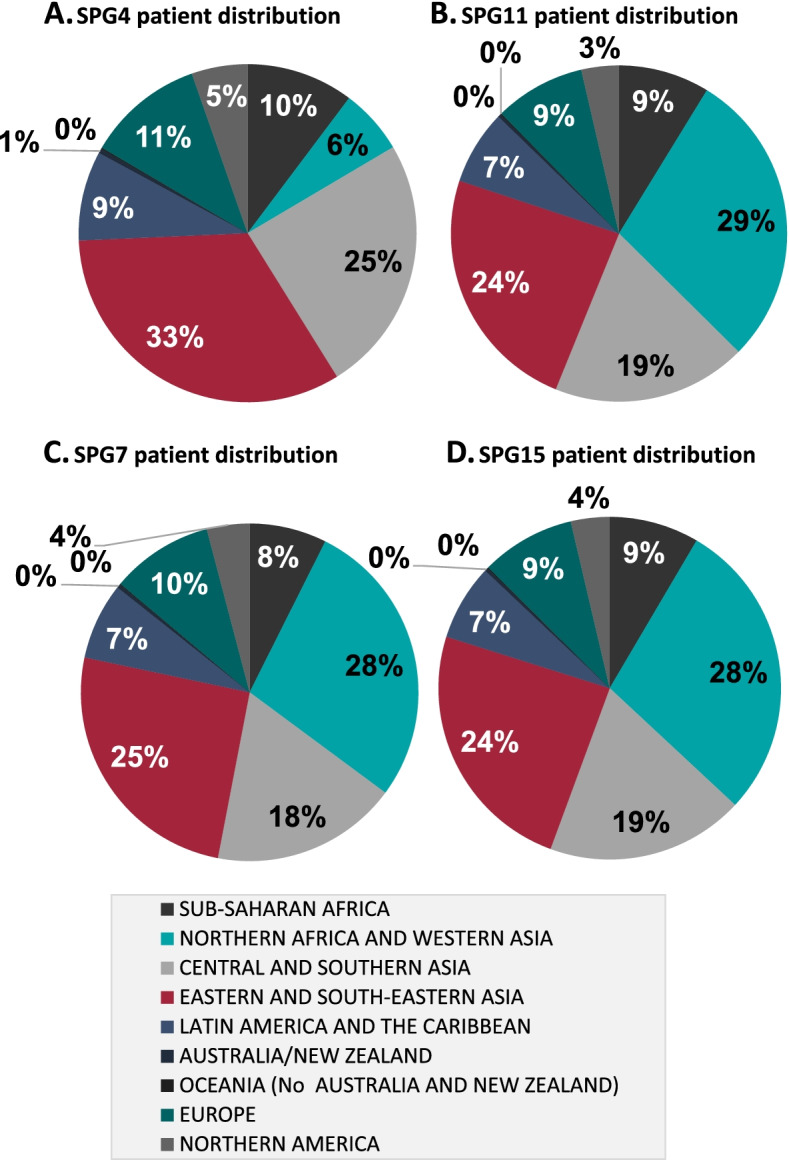
Worldwide distribution of symptomatic patients per genetic subtype. Worldwide estimated distribution of SPG4 (**a**), SPG11 (**b**), SPG7 (**c**), and SPG15 (**d**) symptomatic patients, divided by UN regions: Sub-Saharan Africa, Northern Africa and Western Asia, Central and Southern Asia, Eastern and South-Eastern Asia, Latin America and The Caribbean, Australia/New Zealand, Oceania (no Australia and New Zealand), Europe, and Northern America

For the different genetic subtypes, the estimated global prevalence per 100,000 and the estimated global and regional patient pools were calculated. For SPG4, the estimated global prevalence was 0.90 per 100,000, and the estimated global patient pool was 70,320, which includes 33% of patients in Eastern and South-Eastern Asia; 11% in Europe; and 5% in North America (Fig. 5a). For SPG11, the estimated global prevalence was 0.34 per 100,000, and the estimated global patient pool was 26,839, with an estimated 29% of patients in Northern Africa and Western Asia; 9% in Europe; and 3% in North America (Fig. 5b). For SPG7, the estimated global prevalence was 0.22 per 100,000, and the estimated global patient pool was 16,793, with an estimated 28% of patients in Northern Africa and Western Asia; 10% in Europe; and 4% in North America (Fig. 5c). Finally for SPG15, the estimated global prevalence was 0.13 per 100,000, and the estimated global patient pool was 10,318, with an estimated 28% of patients in Northern Africa and Western Asia; 9% in Europe; and 4% in North America (Fig. 5d).

**Fig. 6:**
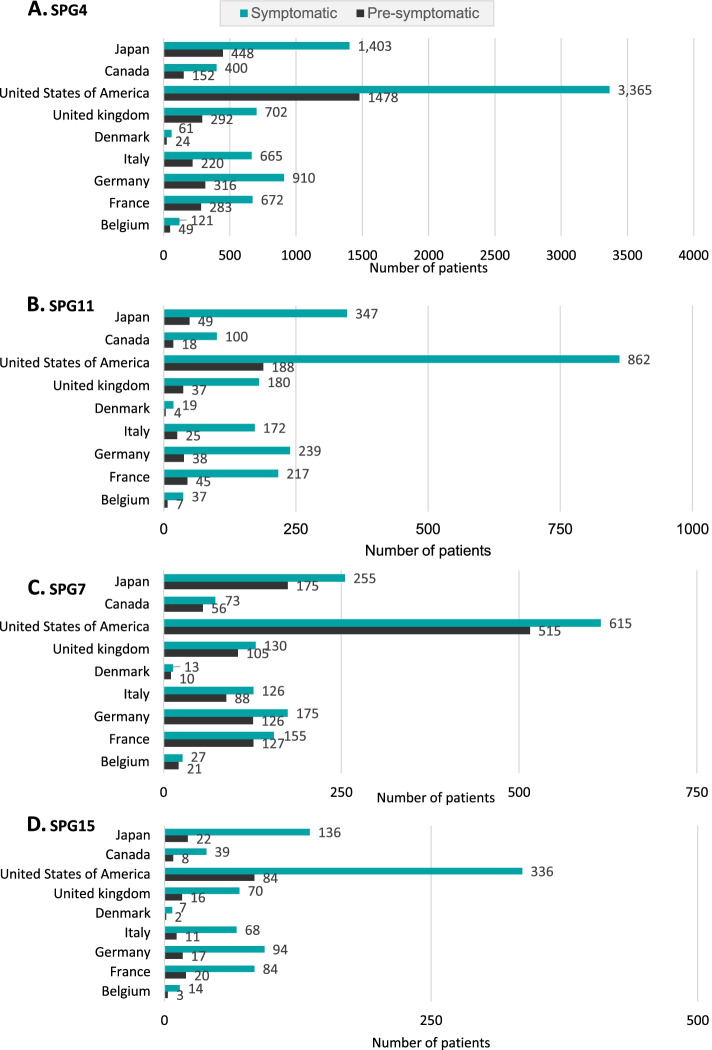
Modelled symptomatic and pre-symptomatic patient pool per country of interest, and per HSP genetic subtype. SPG4 (**a**), SPG11 (**b**), SPG7 (**c**), and SPG15 (**d**). Pre-symptomatic (black) and symptomatic (green) populations are highlighted

In order to estimate the HSP Patient Pool, by key country and genetic subtype, the total number of symptomatic patients was calculated as 124,270, globally. For the key countries studied, and the mutations chosen for this study, the overall total numbers of symptomatic patients were 8298, 2199, 1569 and 859 for SPG4, SPG11, SPG7, and SPG15, respectively (Fig. 6). In line with previous studies, and with the observed late age at onset in HSP, the majority of the symptomatic patients were found to be adults. This is especially the case for SPG4 and SPG7, where the average years living without symptoms were calculated as 24.7 and 36.8, respectively. On average, for SPG11 and SPG15 the symptoms started in the teenage years, with around 13.0 and 14.6 years lived without symptoms, respectively.

## Discussion

The limited availability and high variability of epidemiological HSP data impede an exact determination of incidence, prevalence, and survival. Despite these limitations, available published data based on diagnosed patients allowed for an approximation of the overall HSP patient distribution according to genetic subtype, symptom status, age category, and geographic region. For four of the most common identified genetic subtypes - SPG4, SPG7, SPG11, and SPG15 - data on prevalence, mutation frequency, and age at onset were available, and were used to estimate incidence at birth and survival rates for each genetic subtype. In line with available evidence, adjustments were made for country and ethnic-specific differences in incidence and prevalence. In addition, a more comprehensive quantification of prevalence per region and genetic subtype allowed us to estimate the distribution by symptom status and age category per genetic subtype.

There were several limitations to this study. As with any epidemiological model, it is only as accurate as the underlying evidence. In HSP, like other rare diseases, high-quality evidence is scarce, fragmented, and variable in general and across genetic subtypes. Additionally, certain evidence may reflect bias due to inherent differences in genotyping methods, study design, and ethnic background of the patient population assessed. Nevertheless, the best available evidence in HSP was sought to inform this model. Furthermore, the heterogeneity in patient identification, genetic testing, and diagnosis rates among countries was not considered in the use and interpretation of the various data sources. A limitation of the modelling approach was the use of mean data, which cannot fully account for heterogeneity, such as bimodal distribution of age at onset [5]. To account for this variability in age at onset, a more detailed methodology could be considered in a next step, which applies age at onset, survival, and time to diagnosis probability curves. This would lead to a more accurate estimate of patient distribution by age and by clinical manifestation (pre-symptomatic and symptomatic). An additional limitation were the assumptions made for modifying factors, such as incidence risk with a founder effect and impact of consanguinity in certain populations.

The sensitivity of our model should be considered. Given the limitations of evidence in rare disease, assumptions must be made. Some of the assumptions could have a material impact on the results, such as age at onset variability considering co-variation with incidence at birth. Additionally, the assumptions on the relative incidence ratio of sub-populations (e.g., consanguinity factor applied for Arabic subpopulations) also have an influence on study results. A formal sensitivity analysis was not performed. However, a simulation module was developed in the model to allow for rapidly adapting key assumptions with new and evolving insights. It is relevant to consider that the base-case assumptions and results were assessed by HSP experts and deemed realistic.

To validate the results generated by the model, the data was compared with current epidemiological estimates and assessed by HSP experts. Using the model output, HSP global prevalence was estimated to be 3.6 per 100,000 (3.0–4.3), which was obtained by combining SPG4 or SPG11 global prevalence with their respective fraction of HSP prevalence from Omidvar et al. [1]. This estimate is consistent with the meta-analysis performed by Ruano et al. [2], estimating HSP prevalence to be 3.6 per 100,000 (2–5.3), and slightly above the prevalence of 2.24 cases per 100,000 inhabitants identified in Spain [13]. Additionally, no specific inconsistencies were raised by HSP experts in the relative global prevalence per genetic subtype.

Several contextual factors were considered. Initially a factor impacting age at onset was applied to Asian populations with SPG11. But this was removed based on the high data heterogeneity and high uncertainty around the estimated proportion (CI 0.06–0.99) [31, 36, 40–42]; HSP Experts agreed with the removal of this modifying factor. In line with the older age at onset of HSP and the subjectivity of symptoms, especially in mild cases, unreported cases may have a material impact on the underlying evidence. Based on recent data from France [5], the prevalence of SPG4 alone could potentially be as high as 3.7/100,000. Additionally, there are other regions in which founder effects may apply. For example, there is evidence of a potential founder effect for SPG7 identified in Quebec, Canada [9]. But no founder effect risk factor related to SPG7 variant p.(Ala510Val) was applied in the model due to unclear genotype-phenotype correlation of this variant, as real cases do not match with expected, based on the reported carrier frequency. Although the factor representing consanguinity was applied only to Arabic subpopulations, there are other subpopulations known to have high rates of consanguinity, such as Indian and Pakistani [39]. By also applying the consanguinity factor to these ethnic subpopulations, the prevalence could be even higher for AR-HSP in countries with significant immigrant populations from these regions.

## Conclusions

This is the first reported epidemiological model to describe HSP prevalence at the genetic subtype level globally, as well as at the region and country-level. Given the limited available epidemiological evidence on HSP, this study offers additional data to better capture the burden of illness of this disease. Moreover, improving awareness for HSP and the increasing availability of low-cost genetic testing could further increase reported prevalence numbers. The findings can be used to inform public health policy and healthcare service planning, especially in regions where HSP is estimated to be more prevalent, such as Asia and Northern Africa.

## Supplementary Information

**Additional file 1.**

## Data Availability

The key model input data is made available in Supplementary Table 1. The Excel-based epidemiological model remains the intellectual property of Dynacure.
