# Supplementary material for: An integrated modelling methodology for estimating global incidence and prevalence of hereditary spastic paraplegia subtypes SPG4, SPG7, SPG11, and SPG15
Source: BMC Neurol. 2022 Mar 24;22:115. doi: 10.1186/s12883-022-02595-4 (PMC8944001; doi:10.1186/s12883-022-02595-4)
Supplement: Supplementary file 1 — Additional file 1. [file 12883_2022_2595_MOESM1_ESM.pdf]

Stichele et al. An integrated modelling methodology for estimating global incidence and prevalence of hereditary spastic paraplegia subtypes SPG4, SPG7, SPG11, and SPG15

Supplementary Material

Supplementary Table 1. Key model assumptions

Supplementary Table 2. Evidence overview

Supplementary Table 3. Ethnic composition per UN region

Supplementary Table 4. Ethnic composition per country of interest

Supplementary Table 5. Prevalence study details

Supplementary Table 6. Mean age at onset evidence per genetic subtype

Supplementary figure 1. Flowchart of study inclusion

Supplementary Table 1. Key model assumptions

**Incidence at birth per 100k**	SPG4	1.24 [a]
	SPG11	0.35 [b]
	SPG7	0.34 [c]
	SPG15	0.14 [c]
**Modifying factor: Founder effect**	Relative incidence ratio applied to SPG4	10
	Risk factor assigned to each country/region	2%
**Modifying factor: Consanguinity**	Relative incidence ratio applied to SPG7, SPG11, and SPG15	10 [d]
	Risk factor assigned to each country/region with an Arabic population	Refer to Supplementary table 4 & 5
**Age at onset**	SPG4	24.8 [1]
	SPG11	13.1 [1]
	SPG7	37.2 [1]
	SPG15	14.7 [1]
	SPG4 - Caucasian	21.88 [1]
	SPG4 - Asian	28.85 [1]
	SPG4 - American	30.97 [1]
**Mortality hazard ratio, SPG4 & SPG7**	All ages	1.00
**Mortality hazard ratio, SPG11 & SPG15**	Age: 0 – 35 years	1.00
	Age: 40 – 44 years	1.20
	Age: 45 – 49 years	1.40
	Age: 50 – 54 years	1.60
	Age: 55 – 59 years	1.80
	Age: 60+ years	2.00

[a] *Calculated using weighted average prevalence from [16] and [17]*
[b] *Calculated using prevalence from [17]*
[c] *Calculated using relative mutation frequency (SPG7 to SPG11 & SPG15 to SPG11) from [1]*
[d] *halved for Arabic immigrant populations in non-arabic countries*

Supplementary Table 2. Evidence overview

Study	Country	Prevalence									Patient Distribution				Age at onset			
		Prevalence									Mutation frequency				Age at onset			
		Sporadic	AD	AR	Total	By age	SPG4	SPG11	SPG7	SPG15	SPG4	SPG11	SPG7	SPG15	SPG4	SPG11	SPG7	SPG15
Ruano 2014	Meta-analysis	X	X	X	X						X	X						
Chrestian 2016	Canada										X	X	X		X	X	X	
Omidvar 2019	Meta-analysis										X	X	X	X	X	X	X	
Coutinho 2013	Portugal (national)		X	X	X	X	X	X		X	X	X			X	X		
Erichsen 2009	Norway (southeast)	X	X	X	X	X	X	X			X	X						
Filla 1992	Italy (molise region)		X	X	X													
Boukhris 2009	Tunisia		X	X	X													
Braschinsky 2009	Estonia (national)		X	X	X	X	X	X			X							
Hirayama 1994	Japan (National)				X													
Leone 1995	Italy (Valle d'aosta region)	X	X	X	X													
McMonagle 2002	Ireland (North Ir. & Irish rep.)		X															
Polo 1991	Spain (Cantabria region)				X													
Silva 1997	Portugal (Viano Do castelano)				X													
Skre 1974	Norway (Western)		X															
Tsuji 2008	Japan (National)				X													
Werderlin 1986	Denmark (Zealand)				X													
Sridharan 1985	Lybia				X													
Brignolio 1986	Italy (Turin province)	X	X	X	X													
Chen 1968	China (Guam)				X													
Fink 2013	-										X	X						
Ishiura 2014	Japan										X	X						
Liao 2014	China											X						
Martino 2019	-										X				X			
Orsucci 2014	Italy (Pisa town, Pisa province and Tuscany)				X						X	X	X		X	X	X	
Park 2015	Korea																	
Racis 2014	Italy (North-Western Sardinia region (province of Sassari) - insular)		X		X	X					X	X	X					
Schneider 2018	Germany										X							
Schule 2016	Germany and France										X	X	X	X	X	X	X	
Takiyama 2014	Japan										X							
Balicza 2016	Hungary										X	X	X		X	X	X	
Loureiro 2013	Portugal		X								X				X	X		
Lynch 2015	Greece										X		X		X			
Parodi 2018	France														X			
Coarelli 2019	Europe																X	
Choquet 2015	Canada (Quebec)																X	
Carasco-Salas 2020	Spain (Huelva)										X	X	X					
Stevanin 2008	Multi-country (mainly France)															X		
Hellberg 2019	Sweden				X													

Supplementary Table 3. Ethnic composition per UN-defined regions

UN Region	Base-case	Asian[a]	Founder effect	Arabic
**Sub-Saharan Africa**	98.0%	0.0%	2.0%	0.0%
**Northern Africa & Western Asia**	39.9%	2.1%	2.0%	56.0%
**Central & Southern Asia**	98.0%	0.0%	2.0%	0.0%
**Eastern & South-eastern Asia**	0.0%	97.3%	2.0%	0.7%
**Latin America & Caribbean**	94.0%	0.2%	2.0%	3.8%
**Australia/New Zealand**	89.5%	7.5%	2.0%	1.0%
**Oceania (excl. Australia & NZ)**	98.0%	0.0%	2.0%	0.0%
**Europe**	92.3%	0.8%	2.0%	4.9%
**North America**	94.5%	3.5%	2.0%	0.1%

[a]Modifying factor of Asian ethnicity is maintained in table above as it remained in the model's simulation. In the base-case, it was assumed to impact with a factor of 1 (no effect). 'Arabic' refers to consanguinity factor, which was conservatively applied to the proportion of Arabic subpopulations.

Supplementary Table 4. Ethnic composition per country of interest

Country	Base-case	Asian[a]	Founder effect	Arabic
**Belgium**	92.6%	0.1%	2.0%	5.3%
**France**	90.1%	1.2%	2.0%	6.7%
**Germany**	96.3%	0.3%	2.0%	1.4%
**Italy**	96.3%	0.6%	2.0%	1.1%
**Denmark**	92.5%	0.2%	2.0%	5.3%
**UK**	96.8%	0.8%	2.0%	0.4%
**US**	95.9%	2.0%	2.0%	0.1%
**Canada**	93.0%	5.0%	2.0%	0.0%
**Japan**	0.0%	98.0%	2.0%	0.0%

Supplementary Table 5. HSP prevalence study details

Study	Country (Region)	Population	# Cases	Source of Data	Period Studied	Captured in Ruano, 2014 SLR
Sridharan, 1985	Lybia	519,000	11	Multi	1982-1984	Yes
Brignolio, 1986	Italy (Turin province)	2,327,996	31	Multi	1945-1982	Yes
Polo, 1991	Spain (Cantabria region)	510,000	49	Hospitals + families	1974-1986	Yes
Filla, 1992	Italy (Molise region)	335,211	9	Multi	1979-1990	Yes
Hirayama, 1994	Japan (National)	123000000	109	Hospitals	-	Yes
Leone, 1995	Italy (Valle d'aosta region)	115,270	5	Multi	1981-1991	Yes
McMonagle 2002	Ireland (North Ir. & Irish rep.)	5,436,000	69	Hospitals + families	2000	Yes
Tsuji, 2008	Japan (National)	126,900,000	1103	Registry	2001-2002	Yes
Braschinsky, 2009	Estonia (National)	1,340,000	59	Multi	2004	Yes
Boukhris, 2009	Tunisia	869,700	50	Hospitals	1990-2007	Yes
Erichsen, 2009	Norway (Southeast)	2,633,893	194	Multi	1996-2007	Yes
Coutinho, 2013	Portugal (National)	10,322,000	418	Multi	1994-2004	Yes
Silva 1997	Portugal (Viano Do castelano)	250,061	5	Multi	1994	No
Chen, 1968	China (Guam)	37,975	7	1 Hospital	1960-1966	No
Skre, 1974	Norway (Western)	725,000	31	1 Hospital	1960-1968	No
Werderlin, 1986	Denmark (Zealand)	1,179,000	23	Hospitals	1961-1975	No
Racis, 2014	Italy (North-Western Sardinia region (province of Sassari) - insular)	333,576	67	Multi	2000-2010	No
Orsucci, 2014	Italy (Pisa Town, Pisa province and Tuscany)	1,311,953-20,173,732	45	1 Hospital	2011-2012	No
Loureiro, 2013	Portugal	9,958,333	239	Population-based systematic survey	1993-2004	No
Hellberg 2019	Sweden (national)	9,640,000	235	Registry	2009-2013	No

Multi: overlapping strategies

Families: systematic investigation of families of patients identified via previous sources

Supplementary Table 6. Mean age at onset (Years)

Reference	Region	Population	Number of cases	Not gene-specific	SPG4	SPG11	SPG7	SPG15
**Choquet, 2016**	Canada (Quebec)	N/A	N/A	N/A	N/A	N/A	34.2	N/A
**Chrestian, 2016**	Canada	526	150	N/A	24.6	13.0	33.0	N/A
**Coutinho, 2013**	Portugal	10,322,000	418	N/A	33.0	14.0	N/A	N/A
**Hellberg, 2019**	Sweden	9,640,000	235					
**Omdivar, 2019**	Global (Meta-analysis)	N/A	13,570	N/A	24.8	13.1	37.2	14.7
**Omdivar, 2019**	Caucasian (Meta-analysis)	N/A	N/A	N/A	21.9	13.1	N/A	N/A
**Omdivar, 2019**	Asian (Meta-analysis)	N/A	N/A	N/A	28.9	12.8	N/A	N/A
**Omdivar, 2019**	American (Meta-analysis)	N/A	N/A	N/A	31.0	12.8	N/A	N/A
**Racis, 2014**	Italy (Sardinian)	333,576	67	36.6	N/A	N/A	N/A	N/A
**Orsucci, 2014**	Italy (Pisa town, Pisa Province and Tuscany)	1,311,953-20,173,732	45	37.4	38.7	26.0	39.0	N/A
**Martino, 2019**	Italy	41	21	N/A	28.4	N/A	N/A	N/A
**Lynch, 2015**	Greece	40 families	54	N/A	27.5	N/A	N/A	N/A
**Loureiro, 2013**	Portugal	9,958,333	239	29.9	31.7	N/A	N/A	N/A
**Ishiura, 2014**	Japan	N/A	129	30.0	N/A	N/A	N/A	N/A
**Schule, 2016**	Germany	519 families	608	30.8	35.0	17.0	9.0	N/A
**Balicza, 2016**	Hungary	58	19	28.6	21.0	13.0	37.6	N/A
**Parodi, 2018**	France	N/A	842	N/A	29.3	N/A	N/A	N/A
**Coarelli, 2019**	Europe	N/A	241	N/A	N/A	N/A	35.5	N/A
**Stevanin, 2008**	Global	N/A	76	N/A	N/A	14.0	N/A	N/A

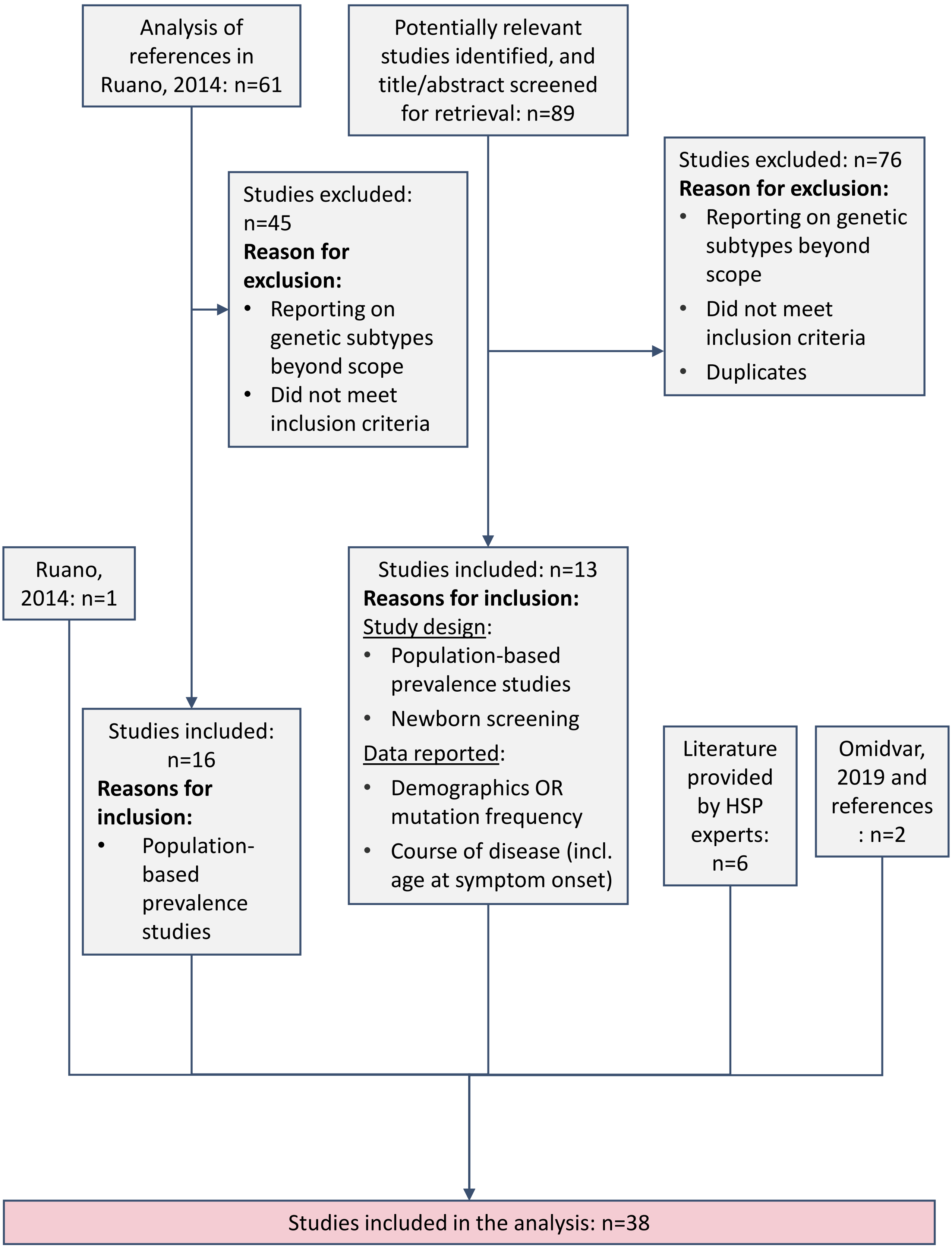

Supplementary Figure 1
